# Supplementary material for: Electronic distance learning satisfaction, awareness, attitudes, and barriers among undergraduate nursing students following the october 7 war in Palestine
Source: PLoS One. 2026 Apr 1;21(4):e0346171. doi: 10.1371/journal.pone.0346171 (PMC13042737; doi:10.1371/journal.pone.0346171)
Supplement: Attachment — Submitted filename: EDL Review Report.docx [file pone.0346171.s001.docx]

**Dear Editor and Authors,**

**I am grateful for having the chance to review this important and timely manuscript. This cross-sectional study represents a very timely examination of undergraduate nursing students regarding their experiences with EDL during the acute crisis following the October 7 war in Palestine. The topic is indeed highly relevant but also satisfies an important gap in the literature concerning education in conflict zones. This well-structured manuscript presents data that is of great value to educational institutions and policymakers. Nevertheless, validity, impact, and overall contribution need several methodological clarifications, interpretative nuances, and contextual framing to strengthen the manuscript.**

**-The abstract is clear on the background, methods, key findings, and conclusions of the study. The critical and interesting finding of the contradiction between positive overall attitude (M=3.02) and overall dissatisfaction (M=2.84) is well-highlighted. To make it even clearer, the abstract should clarify that the cross-sectional design is considered a fundamental limitation to any possible causal inference. The description of the barriers, such as the cost of electricity and internet service, is very good, but the abstract could strongly bring out the fact that the infrastructural deficits were the most salient challenge, which almost dwarfed other aspects of the EDL experience.**

**This introduction effectively sets the global context for E-learning and its accelerated adoption due to crises like the COVID-19 pandemic. The focus on Palestine post-October 7 seems compelling and well-reasoned. For further theoretical and contextual framing, the introduction might more directly reference recent research on the Palestinian educational and psychological landscape. For example, citing Aqtam et al. (2025) regarding the severe psychological consequences of conflict (Psychological impact of political conflict: Prevalence and severity of PTSD among children and adolescents) would powerfully underscore the extreme conditions under which this EDL was implemented, moving beyond a general discussion of conflict to a specific evidence-based context.**

**-The methodology is generally appropriate for the research aims.**

**The descriptive cross-sectional design is suitable for assessing the present state of EDL perceptions.**

**The sample size calculation by using G*Power is clearly reported and appropriate.**

**The use of adapted, previously validated instruments is a strong point. However, the reporting on validity and reliability needs major revision. To state only that a "factor analysis was conducted" is not specific enough; results of this analysis, such as factor loadings, KMO, Bartlett's test, need to be reported to establish construct validity in this unique context. While Cronbach's alpha for attitude and satisfaction are reported, reliability coefficients for the other three domains, Awareness, Preferences, Barriers, are missing and need to be included.**

**_The data collection process through WhatsApp is pragmatic yet should acknowledge the potential for selection bias as such a method could systematically exclude the very students with the most severe connectivity problems.**

**The results are rich in data and presented in comprehensive tables.**

**This is mainly a problem with the interpretation of the Likert scale scores in the results section. It lacks explicit justification for considering a mean of 3.02 ("positive attitude") as only marginally above the neutral point on a 1-5 scale where 3 is "Neutral". Added to this, the "dissatisfaction" score gives a mean of 2.84, which forms a conceptual tension with the former result; such conceptual tension should form part of the explanation of the research findings, but no explanation is given. The authors should clarify whether these scores were contrasted with a theoretical midpoint (for example, 3.0) and justify the chosen cut-off points.**

***Table 2, which outlines attitude items, needs clarification. The labeling of the reversed items as R confused the subsequent classification of the items into "Positive" or "Negative". The table should state clearly whether the mean presented is the final score following reverse-coding.**

**The inferential analyses (t-tests, ANOVAs) are thorough; however, the use of multiple comparisons without a correction (e.g., Bonferroni) does raise the risk of Type I errors. This is something that should be acknowledged as a limitation, or the analyses should be re-run with an appropriate correction.**

**This discussion has done relatively well in comparing findings with other studies from different regions and contexts, such as COVID-19. However, the text will be significantly more powerful with a deeper contextualization of results within the specific reality of the post-October 7 war, using the robust body of work from the region.**

**The section that needs most improvement is the discussion of the attitude-satisfaction paradox. The manuscript should make a clear theoretical proposal. For example, students may hold a pragmatically positive attitude towards EDL as a necessary lifeline for educational continuity (consistent with its utility for "schedule flexibility"), while being dissatisfied with its implementation quality, inability to replicate clinical training, and the immense infrastructural and psychological barriers they faced.**

**Barriers might be discussed by referencing Bsharat et al. (2025, Clinical learning environments and experiences of nursing students in West Bank Universities), which offers a powerful link, suggesting that challenges in the clinical learning environment are now compounded by the barriers of the virtual one.**

**Stress and psychological impact, as discussed here, would be greatly complemented by referring to Aqtam et al. (2025) for an examination of how political violence particularly compounds the more typical stressors that occur in nursing education. This would consequently make findings of the current study evidence-based regarding students' experiences within this particular context.**

**Practical implications are relevant but need to be more concrete instead of a generic appeal for a "blended learning system"; rather, suggest specific strategies such as: "Developing a crisis-responsive blended learning model that includes offline-accessible digital resources to mitigate electricity and connectivity issues, coupled with mandatory psychosocial support services to address the documented trauma and stress among students (Aqtam et al., 2025)."**

**This is an adequate limitations section, but it should be strengthened. The constraints of the cross-sectional design on causal inference and the potential for sampling bias introduced by the WhatsApp-based distribution method are briefly mentioned. The self-reporting bias is touched upon, but could be extended by offering some discussion as to how such biases might interact with the trauma of the ongoing conflict to affect responses uniquely, such as due to social desirability to appear resilient.**

**The reference list is comprehensive with respect to international literature. As explained above, its contextual power would increase considerably by incorporating the recent, highly relevant work of the author group provided here, which would locate this research soundly within the emerging research on Palestinian health and education.**

**Suggested Citations for Contextual Strengthening:**

**Aqtam, I., Ayed, A., Alfuqaha, O. A., & Shouli, M. (2025). Stress and resilience of nursing students in clinical training during political violence: A Palestinian perspective. PLOS ONE, 20(6), e0325278. (To contextualize psychological impact and resilience).**

**Bsharat, N., Aqtam, I., Al-Modallal, H., Dreidi, M., & Shouli, M. (2025). Clinical learning environments and experiences of nursing students in West Bank Universities: A mixed-methods study. PLOS ONE, 20(8), e0327506. (To link findings to the broader context of nursing education challenges in Palestine).**

**Aqtam, I., Naghnaghiyeh, R., Zubaide, T., Othman, Y., & Malasah, Y. (2025). Psychological impact of political conflict: Prevalence and severity of PTSD among children and adolescents in the North and Middle West Bank following the October 7, 2023, events. Nursing Research and Practice, 2025, 7304673. (To underscore the severe mental health environment in which this EDL occurred).**

**In conclusion, this manuscript is about a critical issue and has been conducted with commendable effort under very difficult circumstances. It provides extremely valuable evidence regarding the complex perceptions of EDL among nursing students in a war-torn region. Its methodological underpinnings are present but would benefit from further enhancement through more transparent reporting with respect to the instrument's validity and interpreting the scale scores more subtly. The discussion has the potential to be much more impactful by directly engaging with the specific psychological and educational context of Palestine, drawing upon the excellent local research available. Addressing these major revisions will significantly enhance the manuscript and solidify its status as an important contribution to the fields of nursing education and crisis pedagogy. I recommend major revision and look forward to seeing the strengthened version of this valuable work.**
