# Supplementary material for: Electronic distance learning satisfaction, awareness, attitudes, and barriers among undergraduate nursing students following the october 7 war in Palestine
Source: PLoS One. 2026 Apr 1;21(4):e0346171. doi: 10.1371/journal.pone.0346171 (PMC13042737; doi:10.1371/journal.pone.0346171)
Supplement: Attachment — Submitted filename: Respone PONE-D-25-61436.DOC [file pone.0346171.s002.DOC]

**Electronic Distance Learning Satisfaction, Awareness, Attitudes, and Barriers among Undergraduate Nursing Students Following the October 7 War in Palestine**

Date: Decembar/ 04 /2025

Dear Editor of PLOS ONE,

I would like to thank you for the invitation to review the manuscript entitled “Electronic Distance Learning Satisfaction, Awareness, Attitudes, and Barriers among Undergraduate Nursing Students Following the October 7 War in Palestine ”.

Overall, this is an excellent piece of work that will add significant value to the body of knowledge regarding strategies, such as nursing education and the impact of war on the learning process. It is a well-written manuscript, and I recommend its minor revision. However, I suggest that minor points should be addressed.

**Methods:**

- Describe in further detail why you selected this research design and why it is suitable for your study.
- Add Detailed inclusion/exclusion criteria in separate subheadings.

**Discussion:**

- The implications section is rather short and could be expanded upon by incorporating some of the relevant material appearing elsewhere in the paper.
- Please make the limitations section clearer and highlight more of your study's strengths.
