# Supplementary material for: Electronic distance learning satisfaction, awareness, attitudes, and barriers among undergraduate nursing students following the october 7 war in Palestine
Source: PLoS One. 2026 Apr 1;21(4):e0346171. doi: 10.1371/journal.pone.0346171 (PMC13042737; doi:10.1371/journal.pone.0346171)
Supplement: Attachment — Submitted filename: Review report Distance Learning 2025.DOC [file pone.0346171.s003.DOC]

**Electronic Distance Learning Satisfaction, Awareness, Attitudes, and Barriers among Undergraduate Nursing Students Following the October 7 War in Palestine**

Date: December/ 06 /2025

Dear Editor of PLOS ONE,

Thank you for the invitation to review the manuscript entitled “Electronic Distance Learning Satisfaction, Awareness, Attitudes, and Barriers among Undergraduate Nursing Students Following the October 7 War in Palestine ”.

Thank you for the pivotal and imperative study that is needed in the such conflict zone. The manuscript is well-structured and methodologically sound, but requires minor clarifications. It is a well-written manuscript, and I recommend its acceptance.

*The following points to strengthen the work:*

1**-**The methods and data provide a solid foundation for assessing the impact of Electronic Distance Learning Satisfaction, Awareness, Attitudes, and Barriers among Undergraduate Nursing Students. Explain more why you chose this design and why this Research design is appropriate for your study.

2- The manuscript provides a comprehensive and balanced assessment of existing studies on Electronic Distance Learning.

3- Enhance discussion by incorporating some studies related to the context of Palestinian situation anf factors that may influcne distance learning as well as methods to overcome obstacles that face nursing students in the conflict country.

4- In the result section: What was the final response rate? And how many participants were initially invited?

5- A language revision is required throughout the manuscript to improve readability and clarity.

6- Addressing the suggested revisions will significantly enhance the scientific value and reproducibility of the study.

Sincerely,
