# Supplementary material for: Electronic distance learning satisfaction, awareness, attitudes, and barriers among undergraduate nursing students following the october 7 war in Palestine
Source: PLoS One. 2026 Apr 1;21(4):e0346171. doi: 10.1371/journal.pone.0346171 (PMC13042737; doi:10.1371/journal.pone.0346171)
Supplement: Attachment — Submitted filename: R1 Authors Responses.docx [file pone.0346171.s005.docx]

**Electronic Distance Learning Satisfaction, Awareness, Attitudes, and Barriers among Undergraduate Nursing Students Following the October 7 War in Palestine**

**Responses**

Date: December/ 22/2025

Dear Editor of PLOS One,

I would like to thank you for handling the manuscript entitled "Electronic Distance Learning Satisfaction, Awareness, Attitudes, and Barriers among Undergraduate Nursing Students Following the October 7 War in Palestine". I would like to inform you that we have taken into consideration all the comments.

Response to the Editor’s and Reviewers' comments:

| **Editor comments** | **Authors Responses** |
| --- | --- |
| Dear authors, Thank you for your effort in this manuscript. I hope you can work on the comments provided by the reviewers. Looking forward to see your reply shortly. All the best | Thank you. We have considered all your comments and made the necessary modifications. |
| **Reviewer One comments** | **Authors Responses** |
| This is a timely and highly relevant study. The research addresses a critical, under-researched area the impact of conflict on educational delivery, specifically for nursing students. The descriptive cross-sectional design is appropriate for assessing awareness, attitudes, and barriers. The clarity of the abstract and the summary of key findings are strong. **Areas of Strength** •Timeliness and Relevance: The study is extremely relevant as it focuses on the educational disruptions immediately following the October 7, 2023, conflict in Palestine, providing valuable insights into crisis-time education. •Clear Objectives and Design: The Aim is clearly stated (assessing satisfaction, awareness, attitudes, preferences, and barriers). The Descriptive Cross-Sectional Design is suitable for gathering baseline data on these variables. •Strong Results Section: The abstract clearly presents the major quantitative findings, including the overall positive attitude (M = 3.02) contrasted with the overall dissatisfaction (M = 2.84), which is a compelling result. •Identification of Key Barriers: The study successfully isolated concrete, practical barriers directly related to the conflict/infrastructure (electricity/availability, internet costs, connectivity), providing actionable data for policymakers. •Adequate Sample Size: The sample size calculation (N=288 required, N=292 recruited) demonstrates statistical rigor and addresses the need for adequate power.  **Areas for Improvement and Further Detail 1. Clarity and Consistency of Terminology** •EDLvs. EDL: The manuscript uses "Electronic Distance Learning (EDL)" and "e-learning" interchangeably. While the context is clear, consistent use of a single term (e.g., EDL) throughout the manuscript (title, abstract, methods) is essential for precision. **2. Methods Section Enhancements** •Detailed Tool Description: While the abstract mentions a "valid and reliable self-administered questionnaire," the methods section needs more detail. Specifically: oSource of Adaptation: Clarify how the instruments from Olum, Atulinda (16) and Nikou and Maslov (17) were adjusted and whether these adjustments necessitated a new pilot for validity. **3. Data Presentation** •Statistical Significance Context: The abstract mentions, "The results showed significant differences in satisfaction scores related to sex, age group, and the type of digital tool used." This is a key finding that should be explained more in the results section, including the direction of the differences (e.g., were females more satisfied? Were mobile users less satisfied than laptop users?). •Attitude vs. Satisfaction Disconnect: The mean scores need to be consistently presented as high, medium, or low. The contrast between a positive attitude (M=3.02) and dissatisfaction (M=2.84) is the most impactful finding. Ensure the discussion fully explores why students might think EDLis flexible (positive attitude) but still be dissatisfied (due to the external barriers). **4. Discussion and Conclusion** •Blended Learning Justification: The conclusion recommends a "blended learning system." The discussion should explicitly link the specific barriers (electricity, internet) to this recommendation. •Policy Implications: Strengthen the direct link between the key barriers (electricity/internet) and specific, actionable recommendations for university administrators, IT specialists, and external humanitarian organizations. | Many thanks for your valuable comments. Regarding to the consistency between using the Electronic Distance Learning (EDL)" and "e-learning”. All e-learning were changed in the text to EDL to maintain the same words in the text. Changes were made and highlighted in yellow color all over the text.  In the instrument section the tools were evaluated by expert panel as described. Regarding the pilot study details, they were added as suggested and highlighted in yellow color.  In concern to the data presentation, we make a clearer signaling and interpretation of the result. Changes were made as suggested and highlighted in yellow color.  In the discussion section, we added a paragraph that discuss the main barriers behind the war condition as suggested. Changed were made and highlighted in yellow color.  As an implication for policy issues in this conflict; a paragraph was added to discuss the main implication for October 7 conflict as suggested. Changes were made and highlighted in yellow color. |
| **Reviewer Two comments** | **Authors Responses** |
| I am grateful for having the chance to review this important and timely manuscript. This cross-sectional study represents a very timely examination of undergraduate nursing students regarding their experiences with EDL during the acute crisis following the October 7 war in Palestine. The topic is indeed highly relevant but also satisfies an important gap in the literature concerning education in conflict zones. This well-structured manuscript presents data that is of great value to educational institutions and policymakers. Nevertheless, validity, impact, and overall contribution need several methodological clarifications, interpretative nuances, and contextual framing to strengthen the manuscript. | A paragraph was added to the introduction section to clarify the gap in the literature concerning EDL in Gaza. The changes were added and highlighted in green color.  Validity of the tools used was added to the instrument section, and a clarification was provided regarding the piloting study and the results of internal consistency and validity. |
| **-The abstract** is clear on the background, methods, key findings, and conclusions of the study. The critical and interesting finding of the contradiction between positive overall attitude (M=3.02) and overall dissatisfaction (M=2.84) is well-highlighted. To make it even clearer, the abstract should clarify that the cross-sectional design is considered a fundamental limitation to any possible causal inference. The description of the barriers, such as the cost of electricity and internet service, is very good, but the abstract could strongly bring out the fact that the infrastructural deficits were the most salient challenge, which almost dwarfed other aspects of the EDL experience. | The result section in the abstract is rewritten to highlight the changes as suggested. Changes were made and highlighted in green color. |
| **This introduction** effectively sets the global context for EDL and its accelerated adoption due to crises like the COVID-19 pandemic. The focus on Palestine post-October 7 seems compelling and well-reasoned. For further theoretical and contextual framing, the introduction might more directly reference recent research on the Palestinian educational and psychological landscape. For example, citing Aqtam et al. (2025) regarding the severe psychological consequences of conflict (Psychological impact of political conflict: Prevalence and severity of PTSD among children and adolescents) would powerfully underscore the extreme conditions under which this EDL was implemented, moving beyond a general discussion of conflict to a specific evidence-based context. | Added as suggested in the introduction section with the same reference that you have been suggested. Changes were made and highlighted in green color. |
| **The methodology** is generally appropriate for the research aims.  The descriptive cross-sectional design is suitable for assessing the present state of EDL perceptions.  The sample size calculation by using G*Power is clearly reported and appropriate.  The use of adapted, previously validated instruments is a strong point. However, the reporting on validity and reliability needs major revision. To state only that a "factor analysis was conducted" is not specific enough; results of this analysis, such as factor loadings, KMO, Bartlett's test, need to be reported to establish construct validity in this unique context. While Cronbach's alpha for attitude and satisfaction are reported, reliability coefficients for the other three domains, Awareness, Preferences, Barriers, are missing and need to be included. | Added to the last paragraph of the instrument section and highlighted in yellow color. |
| _The data collection process through WhatsApp is pragmatic yet should acknowledge the potential for selection bias as such a method could systematically exclude the very students with the most severe connectivity problems. | The use of WhatsApp for data collection, while pragmatic in the study context, may have introduced selection bias by potentially excluding students experiencing the most severe internet or connectivity disruptions. This limitation is particularly relevant in conflict-affected settings, where access to digital resources is uneven. Nevertheless, WhatsApp was selected as it is one of the most widely used and accessible communication platforms among students, allowing data collection to proceed despite movement restrictions and safety concerns. This limitation has been acknowledged, and the findings should therefore be interpreted with caution. Future research should consider incorporating alternative or mixed data collection approaches, including offline methods, to better capture the experiences of students with limited or no connectivity. This point was added to the strengths and limitations section and highlighted in green color. |
| **The results** are rich in data and presented in comprehensive tables. | Thank you |
| This is mainly a problem with the interpretation of the Likert scale scores in the results section. It lacks explicit justification for considering a mean of 3.02 ("positive attitude") as only marginally above the neutral point on a 1-5 scale where 3 is "Neutral". Added to this, the "dissatisfaction" score gives a mean of 2.84, which forms a conceptual tension with the former result; such conceptual tension should form part of the explanation of the research findings, but no explanation is given. The authors should clarify whether these scores were contrasted with a theoretical midpoint (for example, 3.0) and justify the chosen cut-off points. | At first thank you for your insightful comment. Secondly, we agree that a clearer justification was needed for the interpretation of the Likert-scale mean scores. In the revised manuscript, we have explicitly stated that all mean scores were interpreted in relation to the theoretical midpoint of the 5-point Likert scale (3.0), which represents a neutral response. Accordingly, the attitude mean score of 3.02 was interpreted as *marginally positive*, reflecting only a slight deviation from neutrality rather than a strong positive attitude. Similarly, the dissatisfaction mean score of 2.84 was interpreted as indicating *mild dissatisfaction*. Changes were done and highlighted in green color. |
| Table 2, which outlines attitude items, needs clarification. The labeling of the reversed items as R confused the subsequent classification of the items into "Positive" or "Negative". The table should state clearly whether the mean presented is the final score following reverse-coding. | Required changes were added to the text and corrected in the table accordingly. Changes were made and highlighted in green color. |
| The inferential analyses (t-tests, ANOVAs) are thorough; however, the use of multiple comparisons without a correction (e.g., Bonferroni) does raise the risk of Type I errors. This is something that should be acknowledged as a limitation, or the analyses should be re-run with an appropriate correction. | We agree with the reviewer that conducting multiple inferential tests may increase the risk of Type I error. As the analyses were exploratory and aimed at identifying potential group differences, no formal correction for multiple comparisons was applied. This has now been acknowledged as a limitation. Changes made and highlighted in green color. |
| This discussion has done relatively well in comparing findings with other studies from different regions and contexts, such as COVID-19. However, the text will be significantly more powerful with a deeper contextualization of results within the specific reality of the post-October 7 war, using the robust body of work from the region.  The section that needs most improvement is the discussion of the attitude-satisfaction paradox. The manuscript should make a clear theoretical proposal. For example, students may hold a pragmatically positive attitude towards EDL as a necessary lifeline for educational continuity (consistent with its utility for "schedule flexibility"), while being dissatisfied with its implementation quality, inability to replicate clinical training, and the immense infrastructural and psychological barriers they faced.  Barriers might be discussed by referencing Bsharat et al. (2025, Clinical learning environments and experiences of nursing students in West Bank Universities), which offers a powerful link, suggesting that challenges in the clinical learning environment are now compounded by the barriers of the virtual one.  Stress and psychological impact, as discussed here, would be greatly complemented by referring to Aqtam et al. (2025) for an examination of how political violence particularly compounds the more typical stressors that occur in nursing education. This would consequently make findings of the current study evidence-based regarding students' experiences within this particular context.  Practical implications are relevant but need to be more concrete instead of a generic appeal for a "blended learning system"; rather, suggest specific strategies such as: "Developing a crisis-responsive blended learning model that includes offline-accessible digital resources to mitigate electricity and connectivity issues, coupled with mandatory psychosocial support services to address the documented trauma and stress among students (Aqtam et al., 2025)."  This is an adequate limitations section, but it should be strengthened. The constraints of the cross-sectional design on causal inference and the potential for sampling bias introduced by the WhatsApp-based distribution method are briefly mentioned. The self-reporting bias is touched upon, but could be extended by offering some discussion as to how such biases might interact with the trauma of the ongoing conflict to affect responses uniquely, such as due to social desirability to appear resilient.  The reference list is comprehensive with respect to international literature. As explained above, its contextual power would increase considerably by incorporating the recent, highly relevant work of the author group provided here, which would locate this research soundly within the emerging research on Palestinian health and education.  Suggested Citations for Contextual Strengthening:  Aqtam, I., Ayed, A., Alfuqaha, O. A., & Shouli, M. (2025). Stress and resilience of nursing students in clinical training during political violence: A Palestinian perspective. PLOS ONE, 20(6), e0325278. (To contextualize psychological impact and resilience).  Bsharat, N., Aqtam, I., Al-Modallal, H., Dreidi, M., & Shouli, M. (2025). Clinical learning environments and experiences of nursing students in West Bank Universities: A mixed-methods study. PLOS ONE, 20(8), e0327506. (To link findings to the broader context of nursing education challenges in Palestine).  Aqtam, I., Naghnaghiyeh, R., Zubaide, T., Othman, Y., & Malasah, Y. (2025). Psychological impact of political conflict: Prevalence and severity of PTSD among children and adolescents in the North and Middle West Bank following the October 7, 2023, events. Nursing Research and Practice, 2025, 7304673. (To underscore the severe mental health environment in which this EDL occurred).  In conclusion, this manuscript is about a critical issue and has been conducted with commendable effort under very difficult circumstances. It provides extremely valuable evidence regarding the complex perceptions of EDL among nursing students in a war-torn region. Its methodological underpinnings are present but would benefit from further enhancement through more transparent reporting with respect to the instrument's validity and interpreting the scale scores more subtly.  The discussion has the potential to be much more impactful by directly engaging with the specific psychological and educational context of Palestine, drawing upon the excellent local research available. Addressing these major revisions will significantly enhance the manuscript and solidify its status as an important contribution to the fields of nursing education and crisis pedagogy. I recommend major revision and look forward to seeing the strengthened version of this valuable work. | All suggestion were addressed and changes were done and highlighted in green color. |
| **Reviewer Three comments** | **Authors Responses** |
| This research article addresses a very timely and relevant topic related to crisis conditions by examining ‘satisfaction, awareness, attitude, and barriers to EDL among nursing students after the October 7 war in Palestine.’ The topic is highly important and can help fill the gap in the literature regarding education during crisis situations. However, the article in its current form has serious shortcomings in reporting methodology, data analysis, and discussion structure that must be addressed before publication in a reputable international journal. Title and Abstract The abstract presents contradictory results. In one sentence, it mentions that the overall attitude is positive, and in another sentence, it says the mean satisfaction indicates dissatisfaction. This contradiction needs clarification. It is suggested that the interpretation of results in the abstract be clearer. For example, it should state that although the overall attitude toward the idea of EDLwas positive, the practical experience of it during war conditions led to a low level of satisfaction. Methodology The methodology section has several fundamental weaknesses. First, the sampling method is not clearly explained. Was it random, a census, or convenience sampling? This ambiguity casts a shadow over the generalizability of the results. Also, inclusion and exclusion criteria for eligible students are not mentioned, and the response rate is not reported. Given the distribution through WhatsApp, mentioning the number of invitations sent and the number of respondents is critical for evaluating the likelihood of bias. Therefore, the ‘Participants and Sampling’ section should be completely rewritten and include precise details of the sampling method, inclusion and exclusion criteria, and response rate. Another weakness is the lack of operational definitions for variables. How exactly were the concepts of ‘satisfaction,’ ‘attitude,’ and ‘awareness’ measured and with what indicators? A complete list of items for each construct should preferably be provided in an appendix. Additionally, the scoring method and interpretation of the Likert scale, for example, the reason for choosing a cutoff of 3 for positive or negative, needs justification. It is suggested to add a separate section titled ‘Variables and Measurement’ that clearly defines each construct and how it was measured. Regarding the validity and reliability of the instrument, although reliability is mentioned, the validity of the instrument is not adequately reported. How was the ‘adaptation’ process of the instrument carried out? Was exploratory or confirmatory factor analysis conducted to examine the structure of the constructs? Merely mentioning review by an expert panel is not sufficient. Providing details of the content and construct validation process is necessary. Findings The reporting of findings is incomplete. For qualitative variables such as awareness and preferences, only percentages are reported, and it would be better to include the number alongside the percentage in the text. In tables related to mean differences, the exact P value for all comparisons is not mentioned and is only shown as an asterisk, which is unprofessional. Exact p-values should be reported. Also, for post-hoc tests after ANOVA, only the existence of a difference is mentioned, and reporting adjusted P values for each pairwise comparison is necessary. Therefore, the findings should be reported with greater precision, and n values, exact p values, and results of post-hoc tests should be included. Furthermore, greater caution is needed in interpreting the mean attitude, which is very close to the neutral cutoff point and is considered ‘positive.’ Can this result really be called ‘positive’ or ‘neutral to slightly positive’? It is suggested to be more cautious in interpreting this finding and to mention the limitation of using a fixed cutoff point. Discussion The discussion section lacks logical structure. Instead of coherent interpretation of findings within the framework of research objectives, comparisons with other studies are made in a scattered manner. The interpretation is incomplete; for example, why is ‘satisfaction’ low despite ‘overall positive attitude’? This apparent contradiction should be analyzed more deeply in the discussion, with reference to identified barriers and the difference between attitude toward the ‘idea’ of EDLand the practical ‘experience’ of it during war conditions. Some comparisons with studies conducted in completely different conditions may be misleading. Also, for some statistically significant differences such as higher satisfaction among men or mobile users, no explanation or hypothesis is provided. It is suggested that the discussion section undergo fundamental rewriting. The suggested structure could include a summary of key findings, interpretation of main findings in light of barriers and war conditions, selective comparison with studies in similar contexts, possible explanation for demographic-based differences, emphasis on practical implications, and greater clarity regarding limitations. Limitations Although some limitations are mentioned, the inherent design limitations of cross-sectional studies are not mentioned. This study does not show a causal relationship. Additionally, using an online questionnaire during electricity and internet outages could lead to severe selection bias in favor of individuals who were in days or areas with better access. It is suggested that these key limitations be clearly included in the limitations section and their potential impact on results be discussed. Minor Issues There is inconsistency in references. In the text, two different sources are cited with the same number, indicating an error in the order of sources. Also, the statement regarding data availability that states ‘data are available from the corresponding author upon reasonable request’ may not be sufficient for journals that emphasize open data access. Efforts to upload data to a public repository are recommended. Final Summary This article has high potential for publication because it addresses a critical and understudied topic. However, at present, due to serious shortcomings in reporting methodology, analysis, and interpretation of results, it is not at the level of a reputable international journal. My recommendation is MAJOR REVISION. If the authors can completely address the mentioned weaknesses, especially in the methodology, reporting of findings, and discussion structure sections, the article will significantly improve and substantially increase the chance of final acceptance. | All suggested issues were resolved and highlighted in blue color. |
| **Reviewer Four comments** | **Authors Responses** |
| I would like to thank you for the invitation to review the manuscript entitled “Electronic Distance Learning Satisfaction, Awareness, Attitudes, and Barriers among Undergraduate Nursing Students Following the October 7 War in Palestine ”.  Overall, this is an excellent piece of work that will add significant value to the body of knowledge regarding strategies, such as nursing education and the impact of war on the learning process. It is a well-written manuscript, and I recommend its minor revision. However, I suggest that minor points should be addressed.  Methods:  •Describe in further detail why you selected this research design and why it is suitable for your study.  •Add Detailed inclusion/exclusion criteria in separate subheadings.  Discussion:  •The implications section is rather short and could be expanded upon by incorporating some of the relevant material appearing elsewhere in the paper.  •Please make the limitations section clearer and highlight more of your study's strengths. | The rational for selecting this design is mentioned as suggested and highlighted with purple color. Details regarding both inclusion and exclusion criteria were added and highlighted with blue color.  Discussion is revised as suggested.  The policy implication is added and all implication section is expanded.  The limitation section is revised as suggested. |
| **Reviewer Five comments** | **Authors Responses** |
| Thank you for the invitation to review the manuscript entitled “Electronic Distance Learning Satisfaction, Awareness, Attitudes, and Barriers among Undergraduate Nursing Students Following the October 7 War in Palestine ”. Thank you for the pivotal and imperative study that is needed in the such conflict zone. The manuscript is well-structured and methodologically sound, but requires minor clarifications. It is a well-written manuscript, and I recommend its acceptance. The following points to strengthen the work: 1-The methods and data provide a solid foundation for assessing the impact of Electronic Distance Learning Satisfaction, Awareness, Attitudes, and Barriers among Undergraduate Nursing Students. Explain more why you chose this design and why this Research design is appropriate for your study. 2- The manuscript provides a comprehensive and balanced assessment of existing studies on Electronic Distance Learning. 3- Enhance discussion by incorporating some studies related to the context of Palestinian situation anf factors that may influcne distance learning as well as methods to overcome obstacles that face nursing students in the conflict country. 4- In the result section: What was the final response rate? And how many participants were initially invited? 5- A language revision is required throughout the manuscript to improve readability and clarity. 6- Addressing the suggested revisions will significantly enhance the scientific value and reproducibility of the study. | Explanation for choosing the cross-sectional design was added as suggested and highlighted in purple color.  The discussion section was enhanced by adding the suggested comments.  Final response rate was already added to the methodology section.  Language was revised, corrected, and highlighted with red color. |
